# Compressive sensing of functional connectivity maps from patterned optogenetic stimulation of neuronal ensembles

**DOI:** 10.1016/j.patter.2023.100845

**Published:** 2023-09-22

**Authors:** Phillip Navarro, Karim Oweiss

**Affiliations:** 1 Electrical and Computer Engineering Department, University of Florida, Gainesville, FL 32611, USA; 2 Department of Biomedical Engineering, University of Florida, Gainesville, FL 32611, USA; 3 Department of Neurology, University of Florida, Gainesville, FL 32611, USA; 4 Department of Neuroscience, McKnight Brain Institute, University of Florida, Gainesville, FL 32611, USA

**Keywords:** compressive sensing, functional connectivity mapping, two-photon microscopy, optogenetics, sparse connectivity, synaptic plasticity, synaptic connectivity

## Abstract

Mapping functional connectivity between neurons is an essential step toward probing the neural computations mediating behavior. Accurately determining synaptic connectivity maps in populations of neurons is challenging in terms of yield, accuracy, and experimental time. Here, we developed a compressive sensing approach to reconstruct synaptic connectivity maps based on random two-photon cell-targeted optogenetic stimulation and membrane voltage readout of many putative postsynaptic neurons. Using a biophysical network model of interconnected populations of excitatory and inhibitory neurons, we characterized mapping recall and precision as a function of network observability, sparsity, number of neurons stimulated, off-target stimulation, synaptic reliability, propagation latency, and network topology. We found that mapping can be achieved with far fewer measurements than the standard pairwise sequential approach, with network sparsity and synaptic reliability serving as primary determinants of the performance. Our results suggest a rapid and efficient method to reconstruct functional connectivity of sparsely connected neuronal networks.

## Highlights

• Mapping synaptic connectivity is key to understand the neural control of behavior
• Random cell-targeted optogenetic stimulation in a sparse network enables rapid mapping
• Compressive sensing reconstructs individual synaptic strength from evoked responses
• 90% recall accuracy can be achieved in less than 30% of trials needed for the sequential approach

## The bigger picture

Reliable perception and action require timely coordination between the synapses of multiple neuron types. Mapping these connections *in vivo* is important for faithful reconstruction of functional brain networks, which ultimately are essential to understand how neurons control behavior. However, current methods to map synaptic connectivity, particularly *in vivo*, require long experiment durations, tend to be low yield, and are unstable. This is mostly because these methods are invasive and employ sequential and independent neural stimulation. Here, we present CoCoMap, an optogenetics-based mapping approach that uses compressive sensing theory to reconstruct synaptic maps using parallel and independent optogenetic stimulation of multiple neurons simultaneously. Using a large model of real biological neural networks, we show that CoCoMap rapidly and efficiently reconstructs synaptic maps with fewer measurements than standard methods and in regions where sparsity is predominantly present.

## Teaser

Mapping synaptic connectivity is key to understanding the neural control of behavior. Navarro and Oweiss describe an approach to perform this mapping in a network of sparsely connected excitatory and inhibitory neurons. They use cell-targeted optogenetic stimulation to evoke synaptic responses in readout neurons and apply compressive sensing to reconstruct individual synaptic strength and type. This results in accurate and precise mapping of connectivity with a significant reduction in the number of trials needed compared to the standard sequential pairwise approach.

## Introduction

There is no doubt that single neurons compute, for example, via specific spatiotemporal axosomatic and axodendritic integration.^1^^,^^2^^,^^3^^,^^4^^,^^5^^,^^6^^,^^7^^,^^8^^,^^9^^,^^10^ It is widely believed, however, that for the brain to support reliable perception and effective action, rapid coordination among ensembles of neurons with heterogeneous cell types, morphologies, and tuning properties is needed.^3^^,^^11^^,^^12^^,^^13^ This coordination is enabled by highly precise and dynamic synaptic connectivity maps that vary in size, location, and architecture across different brain regions.^14^ To map this connectivity, postsynaptic current responses to depolarizing current pulses delivered to presynaptic terminals must be measured using whole-cell recordings—a technically challenging technique with very low yield and recording instability, particularly *in vivo*. While two-photon (2p) or robotic-guided whole-cell patching^15^ may facilitate this approach, the technique remains extremely slow and cannot be performed repeatedly within a session or across multiple sessions.^16^ This precludes the ability to infer large-scale synaptic connectivity maps, for example, to characterize neural circuit function subserving a specific behavior^17^^,^^18^^,^^19^ or to track synaptic plasticity associated with learning and memory formation.^2^^,^^20^^,^^21^^,^^22^^,^^23^^,^^24^

Recent advances in 2p optogenetics have enabled precise spatial and temporal control of neural activity with single-cell resolution in awake behaving animals.^25^^,^^50^^,^^27^^,^^50^ New high-fidelity opsins with improved kinetic properties coupled with advances in spatial light modulation have made it possible to perform multi-cell stimulation with submillisecond precision.^28^ In parallel, advances in genetically encoded voltage indicators (GEVIs) have enabled imaging subthreshold membrane potentials in multiple neurons with high enough signal-to-noise ratios (SNRs).^29^^,^^30^^,^^31^^,^^32^ Together, this all-optical toolkit has paved the way for building high-resolution synaptic connectivity maps of neuronal circuits in awake behaving animals. Despite these striking advances, the ability to map synaptic connectivity in a large population in a reasonable experimental timescale is still out of reach, as neuronal pairs have to be stimulated and recorded sequentially and independently^33^; because connectivity in many brain areas is known to be sparse, with weak or unreliable synapses (such as the neocortex^34^^,^^35^^,^^36^^,^^37^^,^^38^^,^^39^^,^^40^, but see Hunt et al,^41^ Feldmeyer and Sakmann,^42^ Smetters and Zador,^43^ and Pala and Petersen^44^), most trials will have no evoked membrane response.^34^^,^^45^^,^^46^

**Figure 1:**
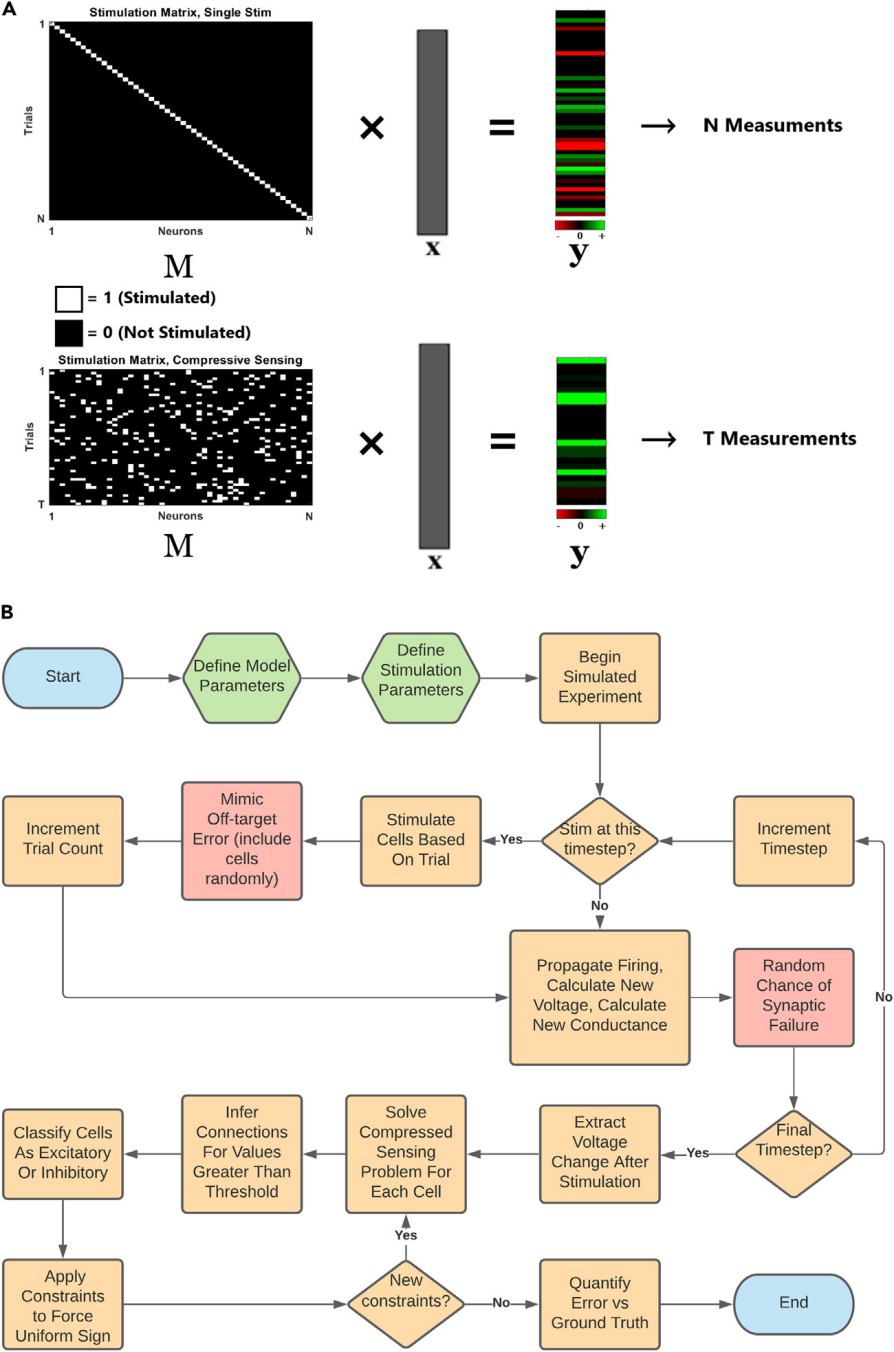
Compressive connectivity mapping (A) Measurement strategies for a system of N interconnected sources. M represents a stimulation matrix delivered to N neurons in the network on each of T trials where a measurement y is made of the recorded neuron in each trial. *x* is a vector of the presynaptic connection weights to the recorded neuron that we wish to estimate. Top: SISO approach based on perturbing one cell in each trial. This takes a number of measurements equal to the number of neurons, with the estimated weights being directly proportional to the measured responses. Bottom: compressive connectivity mapping (CoCoMap) applied to a multiple input and multiple output(MIMO) measurement model. Perturbing multiple neurons per trial and then decoding the mixed responses should enable mapping functional connectivity in fewer measurements. (B) Simulated experiment flow chart. Orange boxes indicate elements that are part of the base experiment. Red boxes indicate steps that only execute if the respective parameter is nonzero. Green boxes indicate parameter selection, and blue boxes denote the beginning and the end of an experimental run. After the model and stimulation experiment parameters are defined, the experiment proceeds by evaluating the differential equations governing the voltage and membrane recovery for each neuron at every time step. At preset intervals, a stimulation trial is carried out that consists of stimulating neurons corresponding to the indices of the trial row in the binary random matrix M. After the final time step, changes in voltage corresponding to the time step after a subset of neurons was stimulated are extracted. These are used to solve the basis pursuit compressive sensing problem 2. Values of *x* greater than some minimum threshold are said to be connections and are compared with the ground truth. Cells are classified as inhibitory and excitatory based on the sign and magnitude of the inferred connections. Projections from these cells are constrained to all be of uniform sign. Error is quantified through metrics of recall and precision.

In this article, we demonstrate a rapid compressed connectivity mapping (CoCoMap) approach that uses parallel random stimulation of presynaptic neurons while measuring evoked membrane postsynaptic potential (PSP) responses from multiple postsynaptic cells simultaneously (Figure 1). This parallel stimulation approach leverages cell-type information, network sparsity, and the theory of compressive sensing (CS)^47^^,^^48^ to map synaptic connectivity using far fewer measurements than the sequential approach. Compared with other related work,^49^^,^^50^^,^^51^ our approach is particularly novel in utilizing cell-type information while also recapitulating much more biologically plausible network topologies and dynamics such as recurrent connectivity, varying levels of background noise, and memory retention of past inputs. We demonstrate that at ∼10% sparsity typically found in cortical networks,^34^^,^^35^^,^^36^^,^^37^^,^^38^^,^^39^ CoCoMap achieved >90% performance with only half the number of measurements that would be needed using the sequential approach. Systematically varying model parameters revealed that CoCoMap outperformed the sequential approach over nearly all examined input and network parameter ranges. CoCoMap performance remained particularly robust in the presence of off-target photostimulation effects and highly clustered small-world network architectures. Observing smaller proportions of the underlying network moderately lowered performance, still resulting in >80% performance with half the measurements needed when only 10% of the population in the network could be observed. Performance plateaued as the fraction of cells in a stimulated pattern increased beyond roughly 20% of the observed population, highlighting trade-offs between the input/output yields in the same trial. For a fixed number of measurements, high synaptic failure probability dramatically lowered performance, suggesting limitations on the approach’s ability to map networks in brain areas known to have unreliable synapses.

## Results

The quality of the reconstruction was evaluated by comparing the estimated connectivity versus the ground truth. Connections correctly determined to exist or not exist were denoted true positives (TPs) and true negatives (TNs), respectively. Error types were defined as follows: a type I error, or false positive (FP), occurred when a connection was declared to exist in the reconstruction but did not exist in the actual model; a type II error, or false negative (FN), occurred when a connection was declared not to exist in the reconstruction but did exist in the actual model. As TPs are of greater interest than TNs for this problem, recall and precision were used as metrics for comparison.

(Equation 1) $$\text{recall}=\frac{\text{TP}}{\text{TP}+\text{FN}}$$

(Equation 2) $$\text{precision}=\frac{\text{TP}}{\text{TP}+\text{FP}}$$

**Figure 2:**
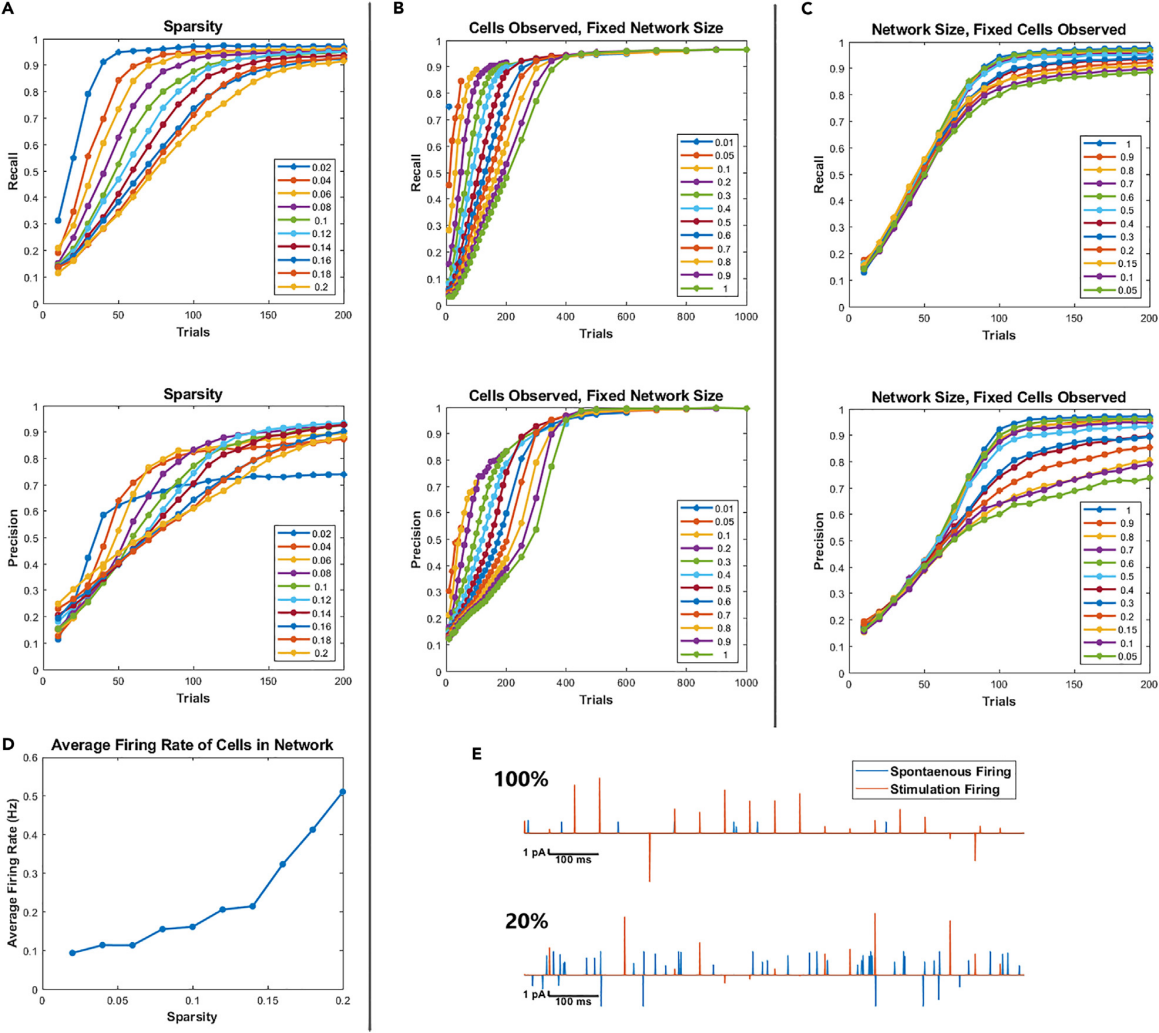
Performance as a function of network size, sparsity, and observability (A) Precision and recall performance as a function of sparsity. Each curve corresponds to a network with a specific probability of connection. (B) Precision and recall performance as a function of the observed population size. The network size was held fixed at 1,000 cells while the observed ensemble size was varied from a fraction of 0.01 (10 cells) to 1 (1,000 cells). T = N trials were performed for each trace. (C) Precision and recall performance as a function of the overall network size. The size of the observed population was held fixed at 200 cells while the total network size was varied from 200 to 4,000 cells. (D) Average firing rates for cells in the network. Increasing connection probability increases background activity. (E) Examples of postsynaptic current events caused by stimulation evoked firing and spontaneous firing for a fully observed and partially (20%) observed network over a 1 s time interval.

At 10% connection probability, we found that CS achieved a >90% recall with only half the trials needed to achieve similar performance using single-cell stimulation (Figure 2A). Similar results were recapitulated at each cell in the network, albeit with higher variance (supplemental information; Figure S2). Higher recall was achieved in fewer trials in sparser networks except for the 2% connection probability case. Because the same regularization parameter, λ, was used for all cases, the sparsity was underestimated in this case, resulting in excessive FPs. CS outperformed single-cell stimulation until the probability of neuronal connectivity rose above 0.16, and then performance fell off (Figure 2A). This drop in performance could be explained by the fact that CS reconstruction generally decreases with decreasing sparsity.^52^ However, due to the partially observed, dynamical nature of the simulated neuronal network, changes in sparsity can contribute to errors beyond those suggested by CS alone. The amount of noise in the system is a function of the background firing, $e_{n_{sf}}$ (Figure 2D), which increases in more densely interconnected networks because the relative contribution of stimulated presynaptic currents becomes small compared to the total network activity in any given trial. Furthermore, as the network becomes more densely connected, more presynaptic inputs are being integrated at their respective postsynaptic cells. This creates larger fluctuations in currents that perturb the membrane voltage significantly, thereby increasing $e_{n_{v}}$ via the nonlinearities in the Izhikevich neuron model used to simulate the network^53^ (supplemental information; Figure S1).

For a fixed network size, we asked what effect the number of observable neurons have on reconstruction performance. Despite the decoding problem growing in complexity as more cells are added, we found that the recall and precision converge to the same points (Figure 2B). We then varied the network size as we held the number of observed neurons fixed. We found that performance dropped as the percentage of neurons observed decreased (Figure 2C). As the percentage of neurons observed decreased, the system became partially observed, and spontaneous firing $e_{n_{sf}}$ began contributing significantly to the measured response (Figure 2E). This suggests that CS may be particularly well suited for models in which observing the entire network is possible, such as *C. elegans*^54^^,^^55^ or zebrafish.^56^

**Figure 3:**
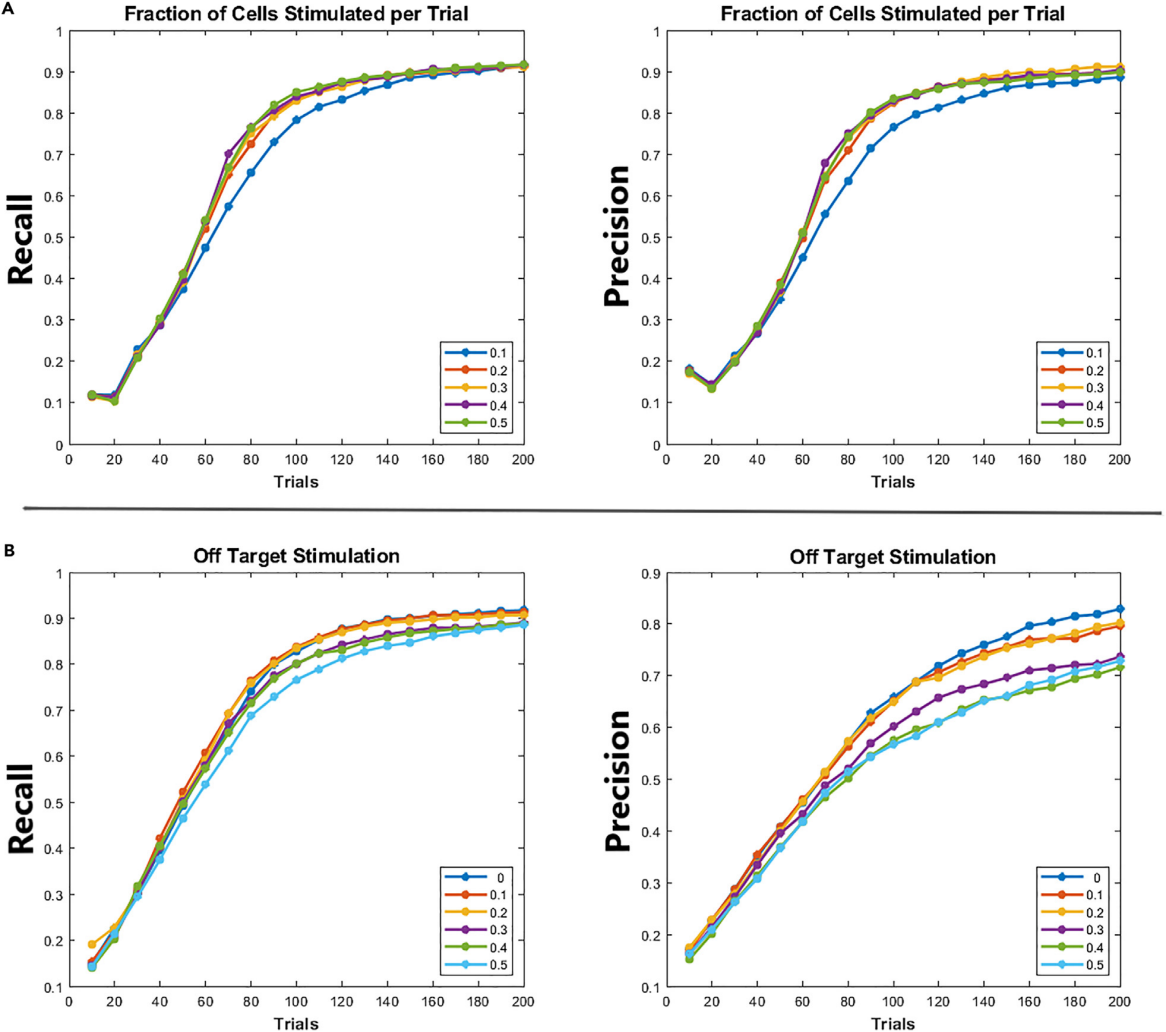
Performance under varying numbers of stimulated cells per pattern and off-target stimulation (A) Precision and recall as a function of neurons stimulated per pattern. Each trace corresponds to the percentage of cells stimulated per trial in the observed population. (B) Precision and recall as a function of off-target stimulation. Each trace corresponds to the probability that an additional neuron per trial was stimulated but not included in the reconstruction.

We then asked whether varying the fraction of neurons simultaneously stimulated as part of a pattern affected the performance. We found that increasing this number beyond 20% of the observed ensemble in a given trial did not lead to substantial gain in performance (Figure 3A, top). This is in contrast to coherence metrics reported in the CS literature, which suggest that performance should increase with increasing nonzero entries in the stimulation matrix.^57^ This deviation could be explained by the dependence of the measured set of neurons on the stimulated set of neurons. Whenever we stimulate a neuron in a trial, we cannot reliably measure the subthreshold response of that neuron in response to depolarization of other presynaptic neurons, as its own depolarization causes a suprathreshold change in membrane conductance. As such, any postsynaptic current it might have received from other stimulated neurons in the same trial would be masked by its suprathreshold response leading to increases in $e_{n_{v}}$ and consequently performance decline.

We then asked whether off-target stimulation had any effect on performance. We found the effect to be marginal (Figure 3B), likely due to the small size and random selection of the off-target neurons relative to the total being stimulated in a pattern. In practice, adequate characterization of laser illumination (e.g., when using computer-generated holography combined with temporal focusing^58^) could limit this effect with knowledge of soma positions in 3D tissue volumes. Furthermore, simultaneous imaging through Genetically encoded calcium indicators (GECIs) and GEVIs^25^ could ameliorate this issue by indicating which neurons fired concurrently with the directly targeted ones. The measurement matrix could then be adjusted post hoc, placing “1”s in trials where cells fired and “0”s where they did not, to facilitate more accurate reconstruction.

**Figure 4:**
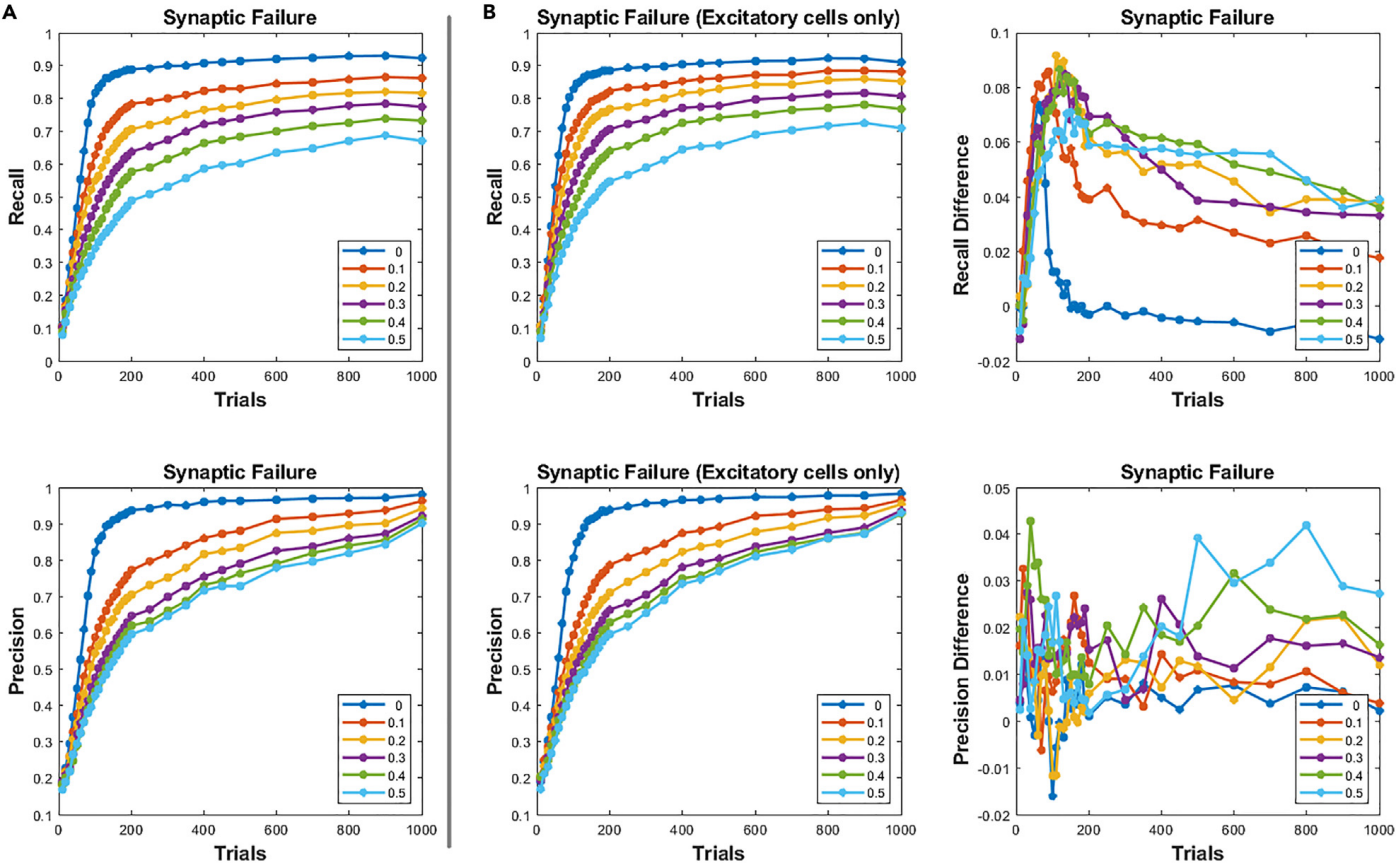
Performance for unreliable synaptic transmission, which also mimics failure to fire a presynaptic neuron, for example, by insufficient laser power/slow opsin kinetics or reduced cell-specific opsin expression (A) Precision and recall as a function of synaptic failure. Each trace corresponds to the probability that an action potential in a presynaptic cell did not propagate to the postsynaptic site. (B) Left: precision and recall as in (A) but for an observed network of only excitatory cells. Right: difference between the two reconstructions in (A) and (B).

We then asked whether synaptic failure had any effect on performance. This could be studied in a multi-input single-output (MISO) system scenario in which only opsins are expressed in a given population while a single postsynaptic cell membrane response is measured at any given time (e.g., using whole-cell patch). In this case, variables such as opsin kinetics, expression level, and laser power could lower the probability of spiking of presynaptic cells (despite the presence of highly reliable synaptic connectivity to the measured cell). We found that recall probability fell dramatically as the probability of synaptic failure increased (Figure 4A). Recall rates of 80% could be achieved but at high failure rates of ∼50% in reconstruction. This resulted from solving an overdetermined system after 1,000 trials for the 200 observed neurons. Although neurons with these rates of synaptic failure have been observed *in vitro*^59^ and *in vivo*,^60^ the mean total network recall rate is on the order of 15%, which suggests that these worst-case simulation results may not be indicative of *in vivo* conditions. If high rates of synaptic failure are encountered, an alternative strategy could be to use cell-type information during stimulation and limit the analysis to specific cell types. In fact, we found that limiting the observed population to only excitatory cells and constraining the reconstruction to specific cell types resulted in a substantial increase in performance (Figure 4B).

**Figure 5:**
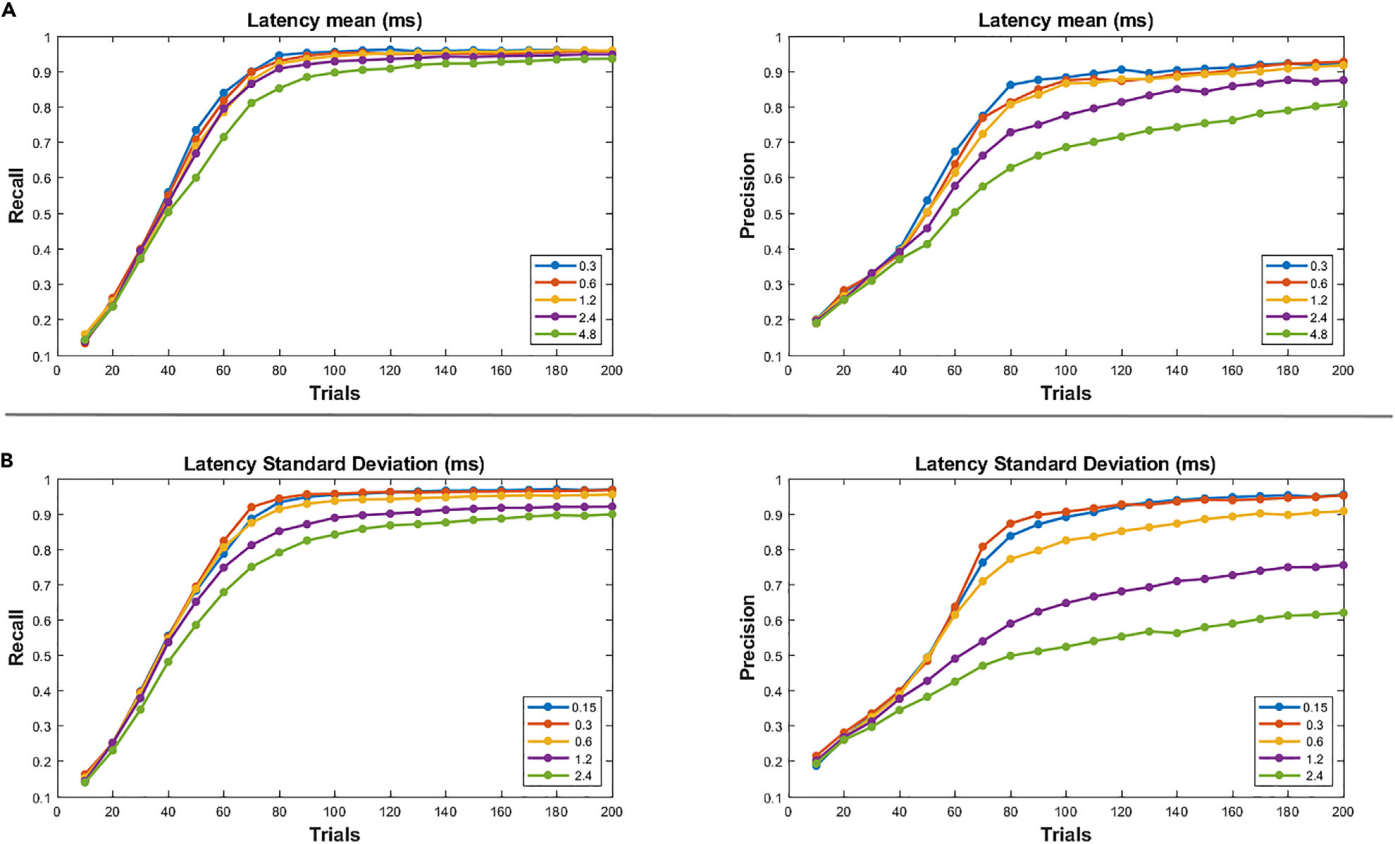
Performance against variable latency The propagation delay for each connection was drawn from a normally distributed random variable with fixed mean and variance. Mean and standard deviation were independently varied while the other variable was held constant. (A) Precision and recall for a fixed 0.6 ms standard deviation with each trace corresponding to a different mean. (B) Precision and recall for a fixed 1.2 ms mean with each trace corresponding to a different standard deviation.

We then asked whether performance was affected by postsynaptic response timing, which could be a function of cell refractoriness, opsin kinetics, and overall brain state. We found that recall probability remained robust as the mean and standard deviation of response latency increased (Figure 5). However, precision declined for mean latencies above 2.4 ms and standard deviations above 1.2 ms. As the mean and standard deviation of latency increase, the interval over which postsynaptic responses must be summed increased. This spreading of the response over a longer interval not only decreases the amplitude of responses relative to the background noise but introduces the possibility of firing from higher-order projections outside of the observed set (polysynaptic effects). However, other studies suggest that this type of noise can be tolerated, as *in vitro* data for pyramidal visual cortex cells had latencies well within the range used here for reconstruction.^61^ Single-cell simulations (Figure S2) also showed good recovery with parameters similar to these data.

**Figure 6:**
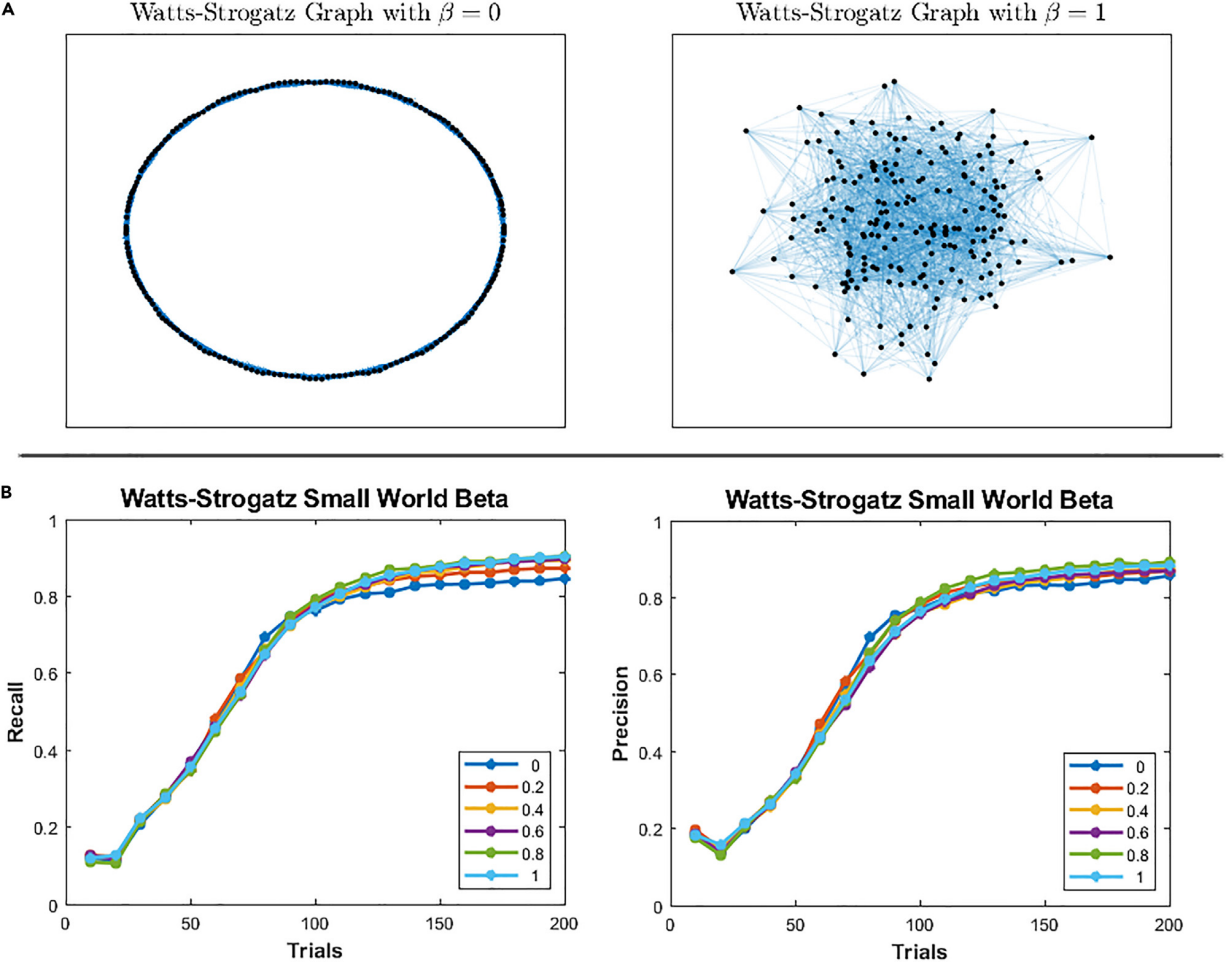
Performance for small-world network topologies (A) Illustrations of Watts-Strogatz graphs for beta values of 0 and 1, where 0 represents a ring lattice structure with highly local connectivity (left) and 1 represents a randomly connected network (right). (B) Recall and precision for small-world beta. Each trace corresponds to networks with a different beta parameter.

We then asked whether network topology had any effect on performance. We found that recall probability remained robust despite varying the small-world clustering characteristics of the network (Figure 6). This may have been a result of the regularity of the degree distribution of the Watts-Strogatz model, where compact support^47^ can be easily satisfied. Models with high variances in degree distributions, such as scale-free networks,^62^ which strain the sparsity requirement of CS with large cliques, may result in poorer performance.

**Figure 7:**
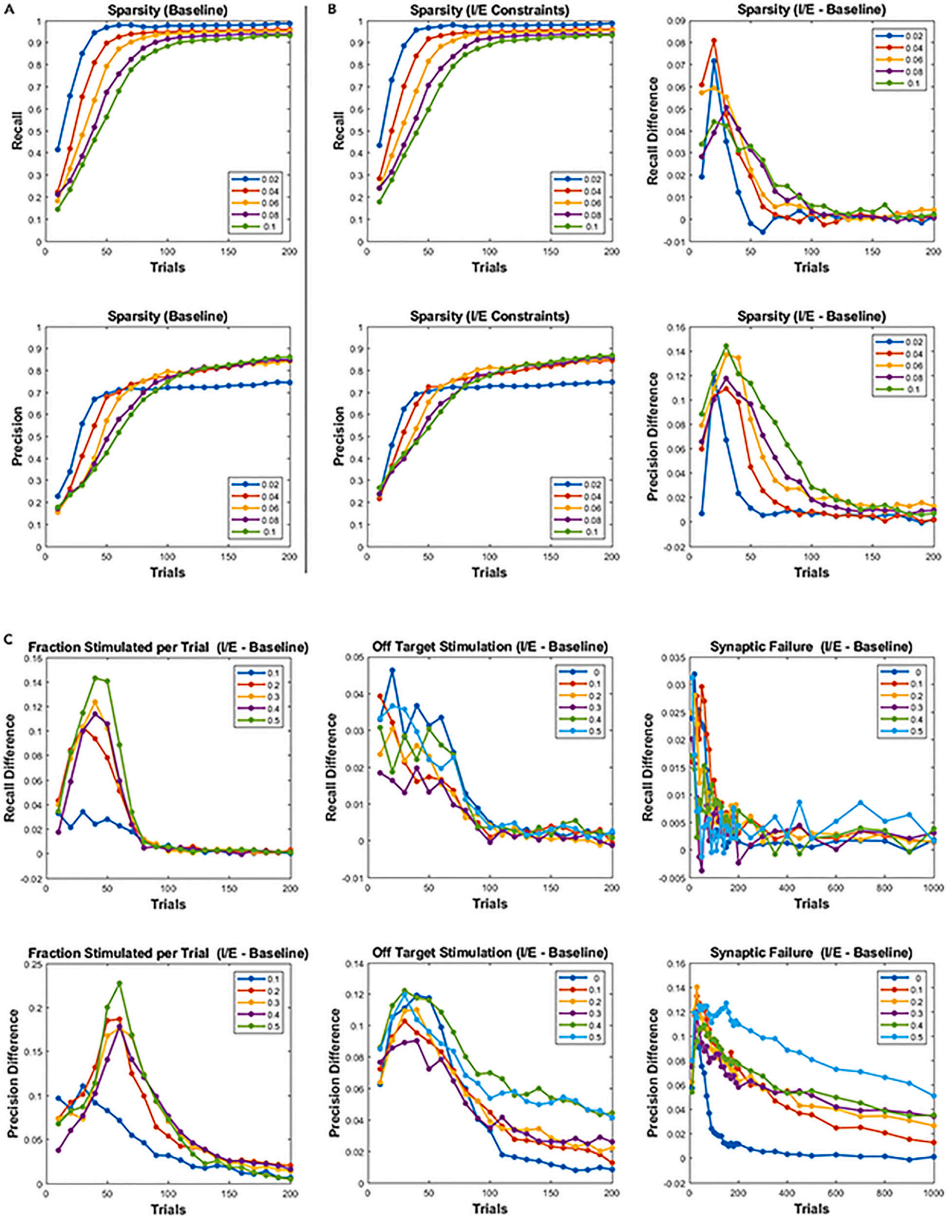
Knowledge of cell type improves connectivity mapping Cells are tagged as inhibitory or excitatory and then forced to take, respectively, negative or positive signed weights during reconstruction. (A) Precision and recall as a function of sparsity. No constraints were placed on cell type during reconstruction. Each curve corresponds to a network with a specific probability of connection. (B) Left: precision and recall as in (A) but with excitatory and inhibitory cells forced to take their respectively signed weights. Right: difference between the two reconstructions in (A) and (B). (C) Precision and recall difference between reconstructions for fractions of cells stimulated per trial, probability of off-target stimulation, and probability of synaptic failure.

We last asked whether knowledge of the observed cell types could improve the performance established above. This is an important experimental design criterion in which cell-type-specific promoters are used in genetically encoded indicators that express in a desired cell type such as pyramidal excitatory or inhibitory subtypes.^63^ We found that adding cell-type constraints improved performance across the full range of tested parameters (Figure 7). Reconstruction with cell-type constraints took fewer trials to achieve the same results as reconstruction without this information. Reconstruction performance plateaued at the same levels with and without cell-type constraints. This is expected, as both cases are converging to the same solution. The plateau falls short of 100%, as recovery can only be guaranteed to within the noise level of the system.^47^

## Discussion

In this work, we explored the use of parallel stimulation and CS for mapping synaptic connectivity between neurons with single-cell resolution, cell-type specificity, and nonlinear dynamics. We constructed a large recurrent network model with parameters derived from biological experiments to quantify multiple aspects of the performance. At predetermined time points, subsets of neurons were stimulated, and PSP responses immediately following the stimulation intervals were measured for each cell. These responses were subsequently decoded by solving a constrained linear programming problem that estimates each presynaptic neuron weight. For each set of parameters tested, recall and precision were computed for the estimated synaptic weights and compared with the ground-truth weights. Results demonstrate that parallel patterned stimulation and CS can be synergistically used to map synaptic connectivity over a wide range of biologically plausible parameters in far fewer trials than sequential stimulation. The wide range of parameters tested also provided further insight into the technique’s limitations.

Model parameters were informed by literature where possible with the goal of recapitulating properties of *in vivo* experiments.^64^ The number of observed neurons in the model was motivated by the average number of neurons typically visible in a 2p optogenetic experiment.^17^^,^^28^^,^^58^ Parameters of the neuron model were randomized over the biophysical range to demonstrate that mapping is robust to variability in neuron parameters. The probability of any cell projecting onto any other cell in the network, i.e., sparsity, was not explored to the full physiological limit. While paired recording connection probability can reach over 50% for certain morphologies of nearby neurons,^50^ compressed sensing’s sparsity assumption was already stressed over the tested range for uniform sparsity. As seen by the success of variable density CS in medical imaging,^65^ denser clusters within a sparse dataset can be handled, but this structure was not explored in this investigation. Synaptic failure spanned the values for measured rates at body temperature layer 2/3 pyramidal neurons in visual cortex.^66^ Latency mean and standard deviation base values were based on recordings taken from pyramidal neurons in visual cortex *in vitro*.^61^

Prior work has applied CS to the synaptic connectivity mapping problem, albeit in highly simplified simulation settings or using postmortem anatomical data.^49^^,^^67^^,^^68^ More recent work has applied this approach combined with parallel stimulation of visually identified cells with measured single-cell postsynaptic currents (PSCs) *in vitro*.^51^ Here, we pushed the limits of the simulation environment by incorporating much larger network models with recurrent connectivity and varying many critical parameters to closely resemble biological networks. Further, we mathematically incorporated cell-type information as constraints on the CS objective function to assess whether this information, typically available in all optical mapping experiments, improves the performance. We chose a membrane voltage-based model for ease of implementation and to mimic a voltage imaging experiment where PSPs from multiple cells are recorded simultaneously. PSCs from whole-cell recordings can be used just as readily as PSPs, by substituting their peak amplitudes into y of Equation 3, as both are measurements proportional to synaptic strength. Prior work^49^ used normalized reconstruction error as the main metric, while our method used recall and precision. We chose to tailor our metric toward experimentalists looking to adopt this method. In the context of using a method for a mapping experiment, patching onto cells declared to be connected is a likely follow-up step.^64^ Whether or not that connection exists would be a better metric to use, as results can be more easily confirmed or rejected.

Our results suggest that combining parallel stimulation and CS is a powerful method for high-resolution connectivity mapping under different scenarios of network sparsity, observability, reliability, and latency. We found that network sparsity and synaptic reliability were primary determinants of the performance. Unreliable synapses greatly degraded performance, particularly when cell-type-specific information was not used. Imposing cell-type constraints during reconstruction improved performance, suggesting that experimentalists should leverage this information when possible. Furthermore, increasing the number of cells stimulated per trial improved reconstruction but plateaued when the average network firing rate reached a certain level that eventually masked the measured PSPs in the decoding step.

The limitations of the modeling framework in our study must be considered. First, a core requirement of CS and the neuron model used is that sources additively combine. In CS, the additive combination of sources gives rise to measurements. In the neuron model, presynaptic inputs are integrated to change the cell’s membrane potential. If this is true, our results should not depend on the neuron model used. However, the modeling framework might not be suitable when these inputs nonlinearly combine, such as in shunting inhibition.^69^ If this issue could be overcome by some means, such as the problem being amenable to a nonlinear extension of CS,^70^ a different neuron model would be needed. While the Izhikevich neuron model simulates spiking dynamics in a computationally efficient way with a plethora of firing dynamics for different cell types, it is incapable of modeling complex synaptic integration and would not be suited for it.

Second, while the partially observable network model recapitulated biological processes of presynaptic spiking probability/synaptic failure, random propagation latency, and variable topology,^71^ it was assumed that these parameters remained constant over the course of a given simulation run. In practice, optically evoked presynaptic spiking and synaptic reliability can be a function of many variables such as intrinsic cell properties,^72^ opsin expression level, time since last spike,^61^ and up/down brain states.^73^^,^^64^

Third, simulation parameters were varied independent of one another in a given simulation run. In practice, they might be interdependent, for example, in a synchronized cortical state.^74^^,^^75^

Fourth, changes in membrane responses were governed by differential equations and constant noise processes in the model. Voltage indicator kinetics were not included in the model, and it was assumed that the measured change in membrane voltage followed the model equations. This made the sum of the integrated PSPs, on average, directly proportional to peak voltage amplitude during the interval immediately following stimulation. In live experiments, however, a matched filtering approach might be required to weigh each time point in the postsynaptic events.^76^^,^^77^

Fifth, off-target stimulation might cause responses from proximal and apical dendrites from other cells, which must be filtered out from the measurement.^76^

Sixth, connections are declared to exist or not based on a comparison threshold determined by the strongest synaptic weight. Because the majority of these weights are small, this predisposes the approach to many FNs. However, a threshold is necessary, as the solution to the CS objective function often contains terms that are very close to zero. Some cutoff is needed, so 1% of the strongest weight was chosen. The voltage change generated by a synaptic weight of this magnitude is on the order of 1/10th of the background noise level of the model. By that metric, we believe this to be a tolerable loss. Lastly, the amount of stimulation needed to evoke a spike was assumed to be known in the simulation. In practice, each cell’s relative response to stimulation is unknown, and these thresholds are found by slowly increasing laser power until the target cell is depolarized.^28^^,^^64^ Errors arising from misestimation of laser power needed to evoke a spike were not modeled. These differences should be accounted for when applying the method *in vivo*.^64^

CS assumes that the measurements taken to perform reconstruction are linear superpositions of components within their respective measurement, and therefore the largest performance decrease occurred when these assumptions were violated. Care must be taken when estimating the sparsity of the network, as an untuned λ, the parameter that controls the trade-off between the number of components in the solution versus the error, can lead to FPs if underestimated and to FNs if overestimated. In practice, underestimation may be preferred, as FPs are much easier to check for with single-cell stimulation than FNs. Checking for FPs would require trials on the order of the number of actual connections, which is much smaller than the number of total possible connections. Reconstructions that constrained neurons to their respective cell types resulted in an increase in performance. Recall and precision fell precipitously as probability of presynaptic spiking/synaptic failure increased. Though this was somewhat ameliorated by limiting the observed network to only excitatory cells and constraining the reconstruction, the applicability of CS-based reconstruction will be limited in areas known to have high levels of unreliable synaptic propagation, such as the hippocampus.^66^ All things considered, our proposed method remains highly promising for a wide range of neural circuits that obey the bounds of high-fidelity reconstruction.^33^^,^^34^^,^^35^^,^^36^^,^^46^^,^^78^ Future work could focus on adapting it to work more accurately in the presence of unreliable synapses and efficient sampling strategies with varying levels of sparsity.

## Experimental procedures

### Resource availability

#### Lead contact

Further information and requests for resources should be directed to and will be fulfilled by the lead contact, Karim Oweiss (koweiss@ufl.edu)

#### Materials availability

This study did not generate new unique reagents.

### CS background

Suppose we wish to find connectivity from N presynaptic cells to a given postsynaptic cell. An experiment is conducted where different sets of putative presynaptic cells are forced to fire while the response of the target postsynaptic cell is measured. Each measurement is generated by the input we provide multiplied by an unknown set of synaptic weights that we wish to determine. If no assumptions are made on the network topology, at least N measurements would be required to avoid solving an underdetermined system of equations, regardless of the stimulation modality used or the number of cells simultaneously stimulated. Generally, an underdetermined system of linear equations has an infinite number of solutions, if any. Thus, the true connectivity is not recoverable from this experiment in fewer than N measurements without further assumptions.

CS gives conditions under which a system of linear equations can be solved in fewer equations than unknowns. These conditions are that the system must be sparse in some domain (time, frequency, wavelet, etc.) and that it be incoherently sampled.^47^^,^^80^^,^^81^ By leveraging the innate sparseness of neuronal connectivity coupled with multi-cell stimulation that targets random subsets of potential presynaptic cells, connectivity could possibly be reconstructed by stimulating multiple cells on fewer trials compared with stimulating single putative presynaptic cells, one in each trial. Imagine an experiment where parallel stimulation of a random subset of neurons in an observable population of N neurons is used to generate T measurements (i.e., trials) of membrane responses (Figure 1A). We define the T × N binary stimulation matrix, M, where in each of the T rows (T < N), a neuron is stimulated if its respective index equals “1” and is not stimulated otherwise. CS theory specifies which structures of M satisfy the incoherence condition.^47^^,^^81^ Running this experiment yields a set of $y_{n}\in R^{Tx1}$ response measurements for each putative postsynaptic neuron *n*, *n = 1*:N. We let $x_{n}\in R^{Nx1}$ represent the unknown weight vector connecting the stimulated neurons to postsynaptic neuron *n*. Let $e_{n}$ be the sum of the spontaneous firing $e_{n_{sf}}$ and the membrane noise $e_{n_{v}}$ of the postsynaptic neuron. Assuming that synaptic currents from stimulated presynaptic neurons are linearly summed (assumption can be relaxed later), our measured response can be expressed as

(Equation 3) $$\begin{array}{c} y_{n}=Mx_{n}+e_{n}\;\text{and}\\ e_{n}=e_{n_{sf}}+e_{n_{v}}\text{.} \end{array}$$

*x* can be estimated from this model by solving

(Equation 4) $$\min\;{\left\|x_{n}\right\|}_{1}\;\text{subject}\;\text{to}\;{\left\|y_{n}-Mx_{n}\right\|}_{2}<\;\varepsilon \text{,}$$

where ${\left\|e_{n}\right\|}_{2}\leq \varepsilon$ is an upper bound on the noise. For a sparse signal and incoherent measurements, which the binary random structure of M satisfies,^47^ the L1 norm minimization of *x* subject to the reconstruction error constraint above yields the fewest number of nonzero presynaptic weights.^82^ This approach, known as basis pursuit,^80^ scales relative to the number of connections per neuron as opposed to single input testing, which scales relative to the total number of neurons in the network (Figure 1A).

### Computational model

In order to test this framework, we developed an *in silico* network model composed of 1,000 Izhikevich model neurons.^53^ The Izhikevich model was chosen for its good balance between biological plausibility, diversity of firing characteristics, and computational simplicity. A detailed description of the neuron model is provided in the supplemental information. For this model, responses are measured as changes in postsynaptic membrane voltages (PSPs) from multiple cells.

The network consisted of a fixed number of observed neurons, N, while varying the total network size so that results could be compared across different levels of network observability. Network parameters were chosen to mimic ratios in mammalian cortex, with neurons being 80% excitatory and 20% inhibitory^83^ (supplemental information S1: neuron model). These neurons were synaptically connected with uniform random probability. Neurons were injected with a zero mean Gaussian random current at each 0.5 ms time step with variance tuned to elicit a 0.2 Hz average spontaneous firing rate per neuron across the network. These parameters were chosen based on reported spontaneous activity in layer 2/3 vS1 barrel cortex and activity in layer 2/3 V1 in awake mice.^34^^,^^50^^,^^78^^,^^84^ A subset of the network was randomly selected for observation to mimic experimental scenarios where only a subset of a large population is observable. A T × N binary random independent identically distributed stimulation matrix, M, was generated. Every 50 ms, a stimulation trial was carried out. This consisted of selecting a row without replacement from M and injecting a current in neurons (with nonzero indices in the row) sufficient to reach each cell’s respective firing threshold. Voltage responses from each observable neuron in the network on the following time step were recorded in each trial. The full process of conducting a simulation run is summarized in the flow chart in Figure 1B.

On top of this base model, we introduced other variables in the model that mimic realistic biological and technological factors. Specifically, synaptic failure was modeled as a binary random variable that sets the amount of current transferred from a presynaptic to a postsynaptic neuron to zero. Off-target stimulation was modeled as an instantaneous current delivered to a random single neuron that is not in the stimulated set in each trial with binary random probability of occurrence. Latency was modeled as a time delay between presynaptic action potential firing and evoked PSC. Specifically, for each connection in the network, latency takes on a value given by a normally distributed random variable rounded to the nearest time step. In models with random latency, voltage responses were measured in a three standard deviation intervals following stimulation. Voltage decay for each cell was estimated by taking the mean of the v(t + 1)/v(t) ratios over all poststimulation intervals. Responses for each time step over these intervals were normalized by subtracting the estimated decay and then summing to obtain the total response for the respective trial.

Local cortical circuits may diverge from uniform sparsity and contain dense clusters of connections,^85^ which could degrade mapping performance. The effect of clustered connectivity was explored by implementing a Watts-Strogatz small-world structure.^86^ The connectivity matrix was constructed by first forming a ring lattice with *n* nodes and *k* edges, depending on sparsity level. A third parameter, beta, controlled the probability of edges’ random reassignment. Beta spanned the range 0–1, with 0 generating a ring lattice of clustered local connectivity and 1 generating a random graph. After the digraph was generated, neurons were assigned to nodes, while weights of the respective cell types were assigned to edges.

### Decoding

The limits of CS were evaluated by varying the model parameters and quantifying map reconstruction using a confusion matrix. Where possible, parameters spanned the range from successful reconstruction to where no reconstruction was possible in fewer trials than the number of neurons. Values for network parameters are based on data from published experiments (Table 1). In each simulated model, each parameter studied spanned its range while other parameters were held at a base value.

**Table 1:** Parameter ranges tested

Experiment parameters, variable in code	Range (base value)^a^
Total neuron in network, TotalCells	200–4,000 (1,000)
Observed neurons, N_obsCell	200–1,000 (200)
Sparsity, sparsity	0.02–0.20 (0.1)
Fraction of neurons stimulated per trial, fractionStim	0.1–0.5 (0.1)
Off-target stimulation probability, offTargetProb	0.0–0.5 (0.0)
Synaptic failure probability, synFailProb	0.0–0.5 (0.0)
Latency mean (ms), latency_mean	0.3–4.8 (1.0)
Latency standard deviation, latency_sd	0.15–2.4 (0.0)
Small-world beta, WSbeta	0.0–1.0 (1.0)
λ, sparsity-error trade-off, lambda	(0.25)

a Each parameter spanned the indicated range while the other parameters were held at the value in parentheses.

The basis pursuit solution 1 was solved for each measured neuron in the network using the CVX modeling system for convex optimization in MATLAB.^87^^,^^88^ A connection was declared to exist if a decoded weight exceeded a threshold relative to the strongest connection observed. An unconstrained version of Equation 4 proved easier to solve in practice (Equation 5):

(Equation 5) $$\min\left({\lambda \left\|x\right\|}_{1}+{\frac{1}{2}\left\|y-Mx\right\|}_{2}\right)\text{.}$$

Here, λ acts as a trade-off parameter between the sparsity of the solution and the fit of the linear reconstruction. Some entries in *x* took values very close to 0, so a cutoff was needed for deciding which were connections. Entries in *x* greater than 1% of the largest weight were declared as true connections.

The objective function was further modified to include knowledge of the cell type, which is typically known in cell-type-specific optogenetic experiments. This was implemented to mitigate the limitation that not all the reconstructed postsynaptic weights were of the same sign. Neurons were classified as excitatory or inhibitory and then added to the optimization as an additional set of constraints of the form

(Equation 6) $$\begin{array}{c} \min\left({\lambda \left\|x\right\|}_{1}+{\frac{1}{2}\left\|y-Mx\right\|}_{2}\right)\\ \text{subject}\;\text{to}\\ x\left(E\right)\geq 0\\ x\left(I\right)\leq 0\text{,} \end{array}$$

where *E* and *I* are the indices of the excitatory and inhibitory neurons, respectively.

## Data Availability

Code for simulations used in this publication is available at https://github.com/phil-navarro/CoCoMap/.^79^

## Supplemental information

Document S1. Figures S1 and S2 and supplemental experimental procedures

Document S2. Article plus supplemental information
