# Supplementary material for: Compressive sensing of functional connectivity maps from patterned optogenetic stimulation of neuronal ensembles
Source: Patterns (N Y). 2023 Sep 22;4(10):100845. doi: 10.1016/j.patter.2023.100845 (PMC10591201; doi:10.1016/j.patter.2023.100845)
Supplement: Document S1. Figures S1 and S2 and supplemental experimental procedures [file mmc1.pdf]

Patterns, Volume *4*

Supplemental information

Compressive sensing of functional connectivity maps from patterned optogenetic
stimulation of neuronal ensembles

Phillip Navarro and Karim Oweiss

Supplemental Items

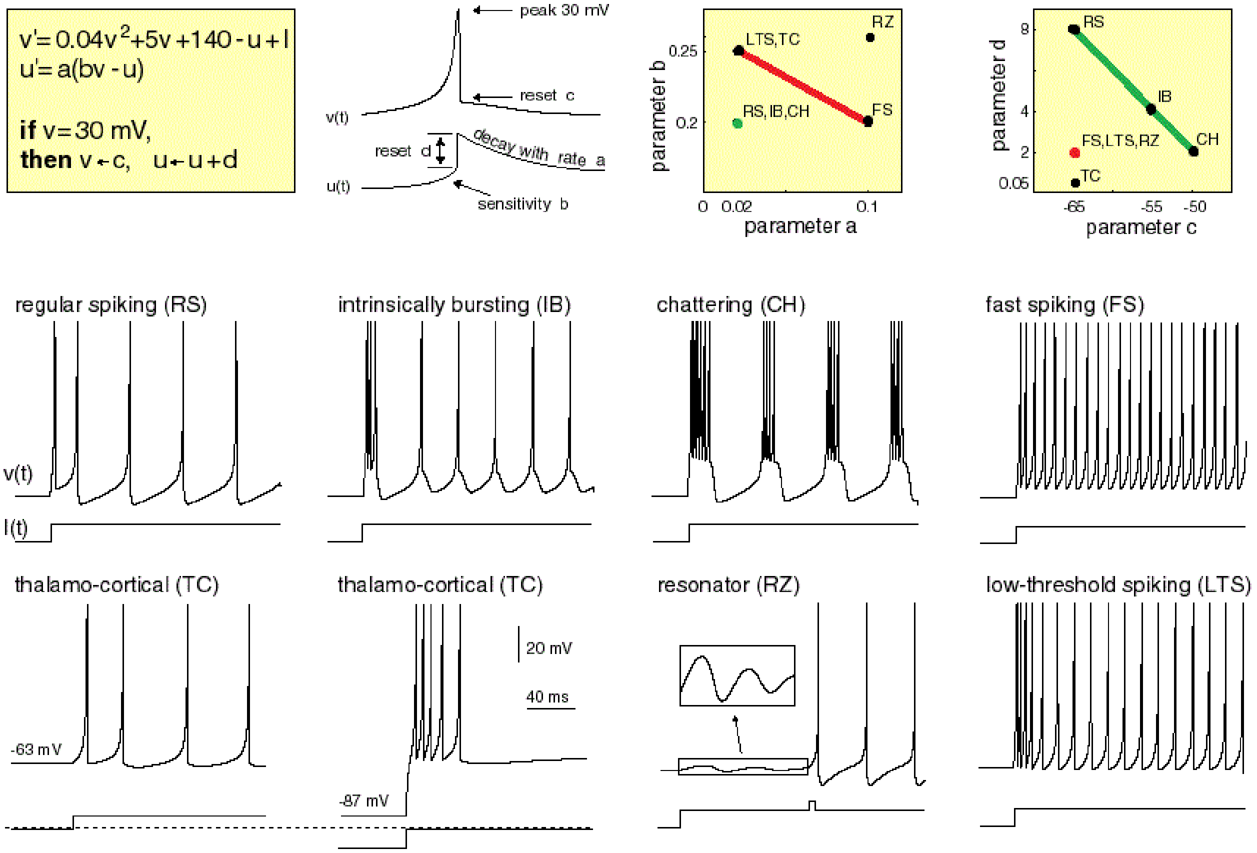

Figure S1. Izhikevich model neurons. Top insets correspond to model dynamics and parameter relationships. Bottom insets correspond to neuron behaviors for different parameter sets. Firing propagates through the model via the 'I' term where 'I' reflects the sum of the weighted connectivity of presynaptic neurons that fired in the previous timestep. Parameter ranges used for excitatory neurons in this proposal are highlighted in green while ranges used for inhibitory neurons are highlighted in red. This modified figure was reproduced with permissions from the author.

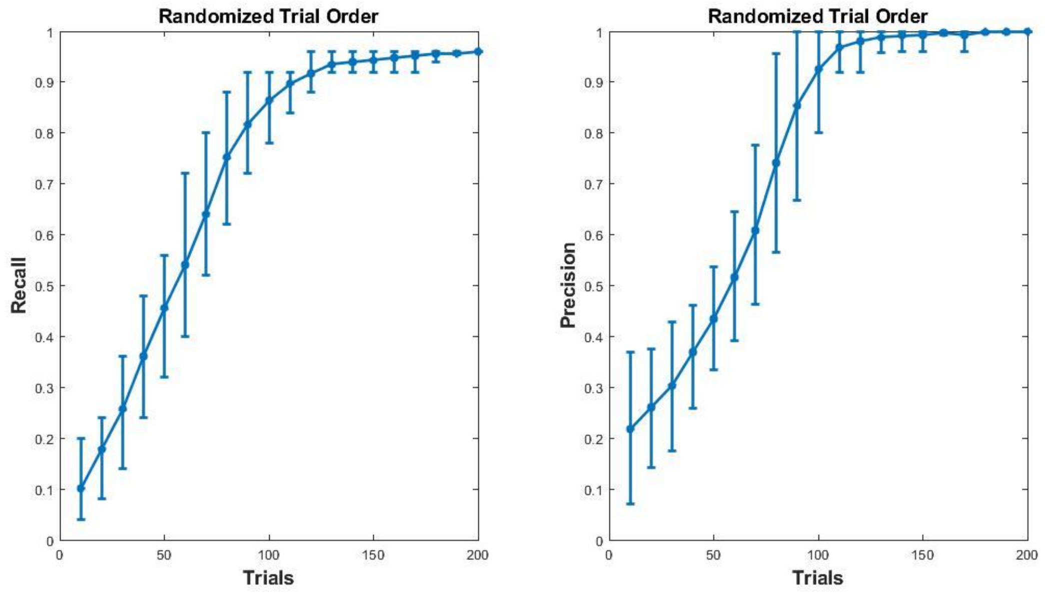

Figure S2. Single cell performance with random trial selection. Recall and precision for 100 randomly sub-sampled reconstructions for neuron 9. Traces correspond to mean performance while error bars span from the 90th to 10th percentile.

Supplemental Experimental Procedures

S1: Single Neuron Model

Spiking neuron models aim to recapitulate firing dynamics of biological neurons. They serve as tools to answer neuro computational questions. The question this investigation wishes to answer is regarding the effectiveness of compressive sensing based functional mapping. The neuron model serves as a tool that takes in input current then outputs a change in voltage, mixed with heterogeneous activity, to be measured and decoded to map connectivity. The neuron model used was the Izhikevich model[1]. It can be described as a biologically plausible model which is computationally simple but capable of producing rich firing patterns for a variety of cell types. It leverages bifurcation methodologies[2,3] to reduce more complex neuronal models to a two-dimensional system of ordinary differential equations:

$$v' = 0.04v^2 + 5v + 140 - u + I$$

$$u' = a(bv - u)$$

With the auxiliary after-spike resetting

$$if\ v \geq 30\ , then\ \begin{cases} v \leftarrow c \\ u \leftarrow u + d \end{cases}$$

Where u and v are dimensionless variables; a, b, c and d are dimensionless parameters; ‘ denotes the derivative with respect to time t. The variable *v* represents the membrane potential of the modeled neuron while u acts as a membrane recovery variable motivated by the biophysical process of potassium ion current activation and sodium ion current inactivation, giving negative feedback to v. Following a spike's peak of 30mV, u and v are reset. The function $0.04v^2 + 5v + 140$ is used for large network simulations[1]. It was derived by fitting cortical neuron spike initiation dynamics so that the membrane potential, v, has mV scale and time t has ms scale[2,4]. Values for the model were obtained by geometrical derivation based on fast and slow nullclines which can be found in[2]. The variable I represents incoming synaptic or injected currents. It is calculated by summing the weights, $S_{i,j}$, of the pre-synaptic neurons, *j*, to post-synaptic neurons, *i,* that fired in

the previous timestep with a zero-mean, normally distributed random input with fixed cell-type specific variance P.

$$I_i = \sum_j S_{i,j} * (v_j \geq 30\,) + N(0, P)$$

The parameter a describes the rate of recovery of u with smaller values resulting in slower recovery. The parameter b couples the membrane recovery u to the membrane potential v with greater coupling leading to dynamics such as subthreshold oscillations and low-threshold spiking. The parameter c corresponds to the reset value of the after-spike membrane potential membrane potential v. The parameter d corresponds to the after-spike reset of the membrane recovery variable u. These parameters typically take values of $(a, b, c, d) = (0.02, 0.2, -65\text{ mV}, 2)$ [1].

Different choices of parameters can lead to neuron behaviors which span what is seen in the brain (Figure S1). RS (regular spiking) neurons respond with a short inter-spike period when first presented with a stimulus then steadily increase in period for the length of the stimulus in a phenomenon known as spike frequency adaptation. Frequency is limited through the voltage reset, $c = -65\text{ mV}$, and large after-spike increase in u, given by $d = 8$. IB (intrinsically bursting) neurons fire a stereotypical burst of spikes when first presented with a stimulus followed by repetitive single spikes. A higher voltage reset, $c = -55\text{ mV}$, and an after-spike increase in u between baseline and an RS neuron, $d = 4$, results in an initial burst followed by a transition into single spiking after u builds up. CH (chattering neurons) fire stereotypical bursts of closely interspaced spikes. A lower after spike increase in u, , $d = 2$, and higher voltage reset, $c = -50\text{ mV}$, make these neurons act like IB neurons that continue bursting and never transition to single spiking. FS (fast spiking) neurons can fire high-frequency periodic trains of action potentials without any spike frequency adaptation. This is modeled as fast membrane recovery by setting a to a large value, $a = 0.1$. LTS (low-threshold spiking) neurons act like fast spiking neurons but have lower firing thresholds and display spike frequency adaptation. This is modeled by increasing the membrane sensitivity to voltage fluctuations, $b = 0.25$ [1]. We chose to only include parameter choices which modeled behaviors observed in cortex (excluding

thalamo-cortical neurons) as that would likely be the first in vivo area studied due to its proximity to the surface of the brain and the depth limit of multiphoton microscopy. Parameters were randomized over this biologically motivated range to produce a network of heterogeneous neurons. Each neuron was designated as excitatory or inhibitory then randomized over a respective parameter range. Excitatory neurons in the model span parameter ranges between RS (regular spiking), IB (intrinsically bursting) and CH (chattering) neurons. Excitatory neurons had $a = 0.02$ and $b = 0.2$ while (c, d) was drawn uniformly from the line between the points (-65,8) and (-50,2). Inhibitory neurons span the parameter ranges between FS (fast spiking) and LTS (low-threshold spiking) neurons. Inhibitory neurons had $c = -65\ \mathrm{mV}$ and $d = 2$ while (a, b) was drawn uniformly from the line between points (0.02,0.25) and (0.1,0.2).

Relating the neuron model equations in terms of CS measurements equation (1), the stimulated firing corresponds to Mx while the spontaneous firing and membrane dynamics form e. Let the superscript ^{C}, typically the set complement, denote neurons in the network model but not in the observed set, and define M_K as a T x K matrix that takes value '1' if a neuron in the respective column fired in the timestep prior to a measured response. We can then write the formula for y in terms of the model equations evaluated at the trial times as:

$$y_n = Mx_n + e_n = v_n^{'}$$

$$v_n^{'} = Mx_n + 0.04v_n^2 + 5v_n + 140 - u + M_K \begin{bmatrix} x_n \\ x_n^C \end{bmatrix}$$

$$e_n = 0.04v_n^2 + 5v_n + 140 - u + M_K \begin{bmatrix} x_n \\ x_n^C \end{bmatrix}$$

$$e_n = e_{n_v}(v, u) + e_{n_{sf}}(M_K, x)$$

This divides the deviation from a linear summation into two terms: e_{n_v} which is dependent on local membrane states and dynamics, and $e_{n_{sf}}$ which is dependent on the spontaneous firing of neurons projecting onto the measured neuron.

S2: Performance under randomized trial selection

In order to assess the effect of trial order on reconstruction performance, the experiment was repeated using randomly sub-sampled trials. The network was initialized with base values for all parameters (1000 Total neurons, 200 observed neurons, 0.1 sparsity, 10% observed neurons stimulated per trial, λ=0.25, propagation latency of 1ms). For a 10 neuron subset of the observed network, 100 simulations were performed for each level of subsampling. In each simulation, T trials were randomly selected, without replacement, from N total trials to be included in the reconstruction. The effect of trial order was measured using the mean, 10th percentile and 90th percentile of recall and precision at each level of subsampling (Figure S2). For every neuron, the reconstruction converged as more trials were included.

Supplemental References

1. Izhikevich, E.M. (2003). Simple model of spiking neurons. IEEE Transactions on Neural Networks *14*, 1569-1572. 10.1109/TNN.2003.820440.
2. Izhikevich, E.M. (2006). Dynamical Systems in Neuroscience: The Geometry of Excitability and Bursting (The MIT Press). 10.7551/mitpress/2526.001.0001.
3. Izhikevich, E.M. (2000). NEURAL EXCITABILITY, SPIKING AND BURSTING. International Journal of Bifurcation and Chaos *10*, 1171-1266. 10.1142/S0218127400000840.
4. Izhikevich, E.M. (2004). Which model to use for cortical spiking neurons? IEEE Transactions on Neural Networks *15*, 1063-1070. 10.1109/TNN.2004.832719.
